# Extended-spectrum *β*-lactamase (ESβL)-producing *Escherichia coli* in antibiotic-free and conventional chicken meat, Brazil

**DOI:** 10.3389/fmicb.2025.1593887

**Published:** 2025-07-08

**Authors:** Bruna Fuga, Ingrith Neves, Herrison Fontana, Jessica Bispo, Elder Sano, Adriana Cardenas-Arias, Fernanda Esposito, Brenda Cardoso, Susan Ienne, Fábio P. Sellera, Nilton Lincopan

**Affiliations:** ^1^Department of Clinical Analysis, Faculty of Pharmaceutical Sciences, University of São Paulo, São Paulo, Brazil; ^2^One Health Brazilian Resistance Project (OneBr), São Paulo, Brazil; ^3^Department of Microbiology, Institute of Biomedical Sciences, University of São Paulo, São Paulo, Brazil; ^4^Department of Cell Biology, Institute of Biological Sciences, University of Brasília, Brasília, Brazil; ^5^Core Facility for Scientific Research (CEFAP), Institute of Biomedical Sciences, University of São Paulo, São Paulo, Brazil; ^6^Department of Internal Medicine, School of Veterinary Medicine and Animal Science, University of São Paulo, São Paulo, Brazil; ^7^School of Veterinary Medicine, Metropolitan University of Santos, Santos, Brazil

**Keywords:** foodborne pathogens, retail meat, chicken meat, Enterobacterales, genomic surveillance

## Abstract

**Background:**

Contamination of food by antimicrobial-resistant pathogens poses significant risk to consumers and environment, potentially leading to foodborne illnesses, silent colonization, and dissemination of antibiotic-resistant bacteria across geographic borders.

**Methods:**

This study analyzed 32 retail meat samples (12 chicken, 10 beef, and 10 pork) from conventional (CN) and antibiotic-free (AF) production systems in Brazil, assessing WHO bacterial priority pathogens through whole genome sequencing and microbiological methods.

**Results:**

Five broad-spectrum cephalosporin-resistant *Escherichia coli* strains were isolated from AF chicken meat, including four extended-spectrum *β*-lactamase (ESβL) producers belonging to sequence types (STs) ST117, ST443, ST1559 and ST3258, and one pAmpC producer carrying the *bla*_CMY-2_ gene and belonging to ST57. On the other hand, four *E. coli* strains resistant to 3rd generation cephalosporins were identified in CN chicken meat, being three ESβL producers of ST38, ST2179 and ST2040, and one pAmpC producer belonging to ST350. Genes conferring resistance to hazardous heavy metals, disinfectants, and pesticides were identified, whereas virulent potential of *E. coli* ST350 and ST2040 was predicted. Noteworthy, *E. coli* ST38 was genomically related to lineages previously identified in poultry (North America) and polluted environments (Europe), supporting an intercontinental dissemination within a One Health framework.

**Conclusion:**

Our findings reinforce the need for continuous surveillance of WHO critical priority pathogens in the chicken meat supply chain from different production systems.

## 1 Introduction

Critical antimicrobial-resistant (AMR) bacteria exert hazardous effects on the environment or humans via contamination, causing serious economic losses and endangering human and environmental health (Antimicrobial Resistance Collaborators, 2022). Multisectoral aspects, including the overuse and misuse of antimicrobials in human and animal healthcare, agriculture, and livestock, have significantly contributed to the rapid emergence and spread of multidrug-resistant (MDR) bacterial strains (Aslam et al., 2021). Indeed, MDR bacteria have increasingly been documented beyond the confines of human hospital walls, adding an additional layer of complexity to this issue (Aslam et al., 2021; McEwen and Collignon, 2018). Given the interconnected nature of this problem, multidisciplinary approaches under the auspices of One Health have been encouraged to strengthen surveillance and mitigate the dissemination of these clinically important pathogens (Jesudason, 2023).

The 2021–2025 Action Plan on antimicrobial resistance by the Food and Agriculture Organization of the United Nations (FAO) has highlighted five key strategic priorities aimed at addressing this issue within the food and agriculture sectors, including: (i) promoting practices to prevent infections and mitigate the spread of antimicrobial resistance; and (ii) ensuring the prudent use of antimicrobials to preserve their efficacy. Among these priorities, surveillance is the basis for understanding the dynamics of antibiotic resistance in food, enabling the detection of emerging threats, and guiding targeted interventions to mitigate the spread of resistant microorganisms (Keck et al., 2023).

Particularly concerning is the widespread dissemination of third-generation cephalosporin-resistant *Escherichia coli* producing extended-spectrum (ES*β*Ls) and/or plasmid-mediated AmpC (pAmpC) β-lactamases, rendering them clinically ineffective (Kaper et al., 2004; Foster-Nyarko and Pallen, 2022; Fuga et al., 2022). Due to its high mortality rates, healthcare burden, prevalence of resistance, and other significant impacts on public health, this sort of *E. coli* strains have been classified as a critical priority pathogen by the World Health Organization (2024). Therefore, contamination of food by critical priority *E. coli* represents serious a public health concern due to its potential transmission to humans through the food supply chain (Ramos et al., 2020; Alegría et al., 2020; Mughini-Gras et al., 2019). In this regard, chicken meat is a food highly susceptible to contamination by various microorganisms throughout the food chain, leading to its spoilage and risk to human health and the environment.

Currently, the production of antibiotic-free chicken meat has been encouraged as a response to growing concerns about AMR and the potential human health risks associated with the consumption of meat products containing antimicrobial residues (Haque et al., 2020; Mohammadi et al., 2023). AMR surveillance on antibiotic-free (AF) meat has been proposed to evaluate the effectiveness of antimicrobial stewardship practices in reducing the dissemination of antimicrobial-resistant bacteria in the food supply chain (Farooq et al., 2022). Although some studies have demonstrated that AF meat may present lower levels of antimicrobial residues compared to conventionally raised meat (Sarkar et al., 2023), it can still harbor bacteria of clinical interest, including critical priority *E. coli* strains (Rawat et al., 2024).

Since animal-derived foods can be contaminated with a wide variety of hazardous bacteria, such as *E. coli*, the identification and genetic context of pathogenicity and antibiotic resistance is very important for prevention against their widespread, especially MDR strains. In this study, as part of the Grand Challenges Explorations: New Approaches to Characterize the Global Burden of Antimicrobial Resistance Program, we report the occurrence of global clones of WHO critical priority *E. coli* in both conventional (CN) and AF retail meat, in Brazil, one of the world’s leading meat producers and exporters, and a significant player in the global meat industry (Klein and Vidal, 2022).

## 2 Materials and methods

### 2.1 Sample collection

Between August and February 2019–2020, 32 different samples of chicken, bovine and swine meat sold in supermarkets located at all regions of São Paulo, the most populous city in Latin America, were aseptically collected. The meat samples were obtained from CN or AF production systems, and include retail chicken (*n* = 12), beef (*n* = 10) and pork meat (*n* = 10) (Supplementary Table S1). The storage methods of meat samples, both fresh (FS) and frozen (FZ), were also assessed.

### 2.2 Isolation of broad-spectrum cephalosporin resistant *Escherichia coli* from conventional and antibiotic-free labeled commercial meat

Samples were purchased and immediately stored in thermic boxes and processed within 4 h. Microbiological analyses were carried out according to the Food and Drug Administration (FDA) protocol with modifications (U.S. Food and Drug Administration, 2021). In brief, 100 g of each sample were rinsed with 225 mL of Buffered Peptone Water (BPW) in sterile plastic bags (Whirl-Pak; Nasco, WI), and homogenized by hand-massage for 15 min. Subsequently, 25 mL was transferred to 25 mL of MacConkey broth flasks and cultured overnight at 37 °C. Then, 10 μL aliquot were transferred to MacConkey agar plates supplemented with ceftriaxone (2 μg/mL), as previously recommended for screening of potential ES*β*L-producing *E. coli* (Jacob et al., 2020).

After overnight incubation at 37°C, presumptive *E. coli* typical colonies were subculture into eosin methylene blue (EMB) agar and further confirmed by matrix-assisted laser desorption ionization–time of flight mass spectrometry (MALDI-TOF MS) analysis (Singhal et al., 2015).

### 2.3 Antimicrobial susceptibility testing and confirmation of extended Spectrum β-lactamases (ESβL) phenotype

Antimicrobial susceptibility profiles were accessed by disc diffusion method and results interpreted according to the Clinical and Laboratory Standards Institute (2024) breakpoints. A panel of 14 antimicrobials was tested, including amoxicillin/clavulanic acid (AMC), ceftriaxone (CRO), ceftazidime (CAZ), cefotaxime (CTX), cefoxitin (CFO), cefepime (CPM), ertapenem (ETP), imipenem (IMP), meropenem (MER), nalidixic acid (NAL), ciprofloxacin (CIP), aztreonam (ATM), gentamicin (GEN), and amikacin (AMI). Additionally, double disc synergy test (DDST) was used to detect ESβL production (Jarlier et al., 1998; Drieux et al., 2008). Isolates classified as non-susceptible to at least one agent in three or more antimicrobial categories were defined as MDR, according to the criteria established by Magiorakos et al. (2012).

### 2.4 Genome sequencing

Of the 14 strains recovered from the collected meat samples, nine *E. coli* isolates from chicken meat, representing both CN and AF systems, were selected for whole genome sequencing mainly based on their ESβL phenotype, as determined by the DDST test, with additional consideration of their MDR profiles (Table 1).

**Table 1:** Characteristics of sequenced hazardous *Escherichia coli* strains isolated from antibiotic-free (AF) and conventional (CN) chicken meat.

Strain	Source	Storage	ST	Resistome
F1B	AF	Frozen	ST443	*bla*_CTX-M-2_, *bla*_TEM-106_, *tet(A), aac(3)-VIa, ant(3″)-Ia, qnrB19, sul1*
FCC3	CN	Frozen	ST350	*bla*_CMY-2_, *ant(3″)-Ia, aac(3)-VIa, gyrA*-S83L, *gyrA*-D87G*, parC*-S80I*, sul1, sul2*
FBP3	CN	Frozen	ST38	*bla*_CTX-M-2_, *cmlA1, catA1, tet(B), ant(3″)-Ia, aadA2, aph(6)-Id, aph(3″)-Ib, dfrA7, dfrA15, gyrA*-S83L*, gyrA*-D87G*, parC*-S80I*, sul1, sul2*
FCC4	CN	Fresh	ST2179	*bla*_CTX-M-8_, *bla*_TEM-1B_, *tet(A), aph(6)-Id, aph(3″)-Ib, gyrA-*S83L*, gyrA*-D87N*, parC*-S80I*, sul2*
FBC4	CN	Fresh	ST2040	*bla*_CTX-M-55_, *bla*_TEM_, *aac(3)-VIa, ant(3″)-Ia, fosA3, sul1*
FCC8	AF	Frozen	ST3258	*bla*_CTX-M-55_, *bla*_TEM-141_, *tet(B), aac(3)-IV, ant(3″)-Ia, aph(3″)-Ib, aph(3′)-Ia, aph(4′)-Ia, aph(6)-Id, fosA3, sul2*
FCC10	AF	Frozen	ST15579	*bla*_CTX-M-8_, *tet(B), aac(3)-VIa, ant(3″)-Ia, aph(3″)-Ib, aph(6)-Id, sul1, sul2*
FSE11	AF	Frozen	ST57	*bla*_CMY-2_, *tet(A), ant(3″)-Ia, dfrA1, sul1*
FSW11	AF	Frozen	ST117	*bla*_CTX-M-55_, *bla*_TEM-141_, *fosA3, gyrA*-S83L*, gyrA*-D87N*, parC*-E84K

Total DNA of *E. coli* strains was extracted using a PureLink quick gel extraction kit (Life Technologies, CA), and subsequently utilized for constructing a Nextera DNA Flex Library Prep (Illumina Inc., San Diego, CA). Genomic sequencing was performed using the NextSeq platform (Illumina, San Diego, CA).

### 2.5 Bioinformatics analysis

The paired-end reads were quality checked and trimmed (PHRED quality score <20) using TrimGalore v.0.6.7^1^ and assembled by Unicycler v.0.5.0^2^. For the *E. coli* strain FCC4, we conducted both trimming and *de novo* assembly of sequences using CLC Genomics Workbench v.12.0.3 (Qiagen, Hilden, Germany). Complete genome annotation was carried out using NCBI Prokaryotic Genome Annotation Pipeline v.3.2^3^.

Public databases for molecular typing and microbial genome diversity – PubMLST^4^ and Enterobase^5^ was used to determine sequence type of *E. coli* strains. To evaluate antimicrobial resistance genes, we employed the Resfinder v.4.4.2 tool available through the Center for Genomic Epidemiology (CGE) pipeline^6^, with default settings.

ABRicate v0.9.8^7^ was used to predict virulence genes (VirulenceFinder v.2.0), plasmids (PlasmidFinder v.2.1), fimbriae (FimTyper v.1.0), and serotype (SerotypeFinder v.2.0) profiles through the CGE database. The Virulence Factor Database - VFDB^8^ was additionally employed for predicting the virulome. Heavy metal (HM), herbicide (glyphosate), and disinfectants (QACs) resistance genes were also identified using ABRicate through a database constructed from NCBI and BacMet2^9^. Cutoff values of ≥90% identity and ≥ 80% coverage were used. Phylogroup stratification was performed using ClermonTypingv.1.4.0^10^. The Mlplasmids v2.1.0^11^ was used to predict plasmid and chromosome-derived sequences.

### 2.6 Phylogenetic analysis

For phylogenetic purposes, we downloaded all genome assemblies with data for country, year of collection and source of isolation for each of the STs from the *Escherichia/Shigella* database in Enterobase (see text footnote 5). For each ST, the average nucleotide identity (ANI) between *E. coli* strains and the downloaded dataset was obtained using FastANI v1.32^12^, and the 30 genomes with highest ANI were select for phylogenetic analysis. ST3258 had only 18 genome assemblies available, so all genomes were used. CSI Phylogeny v1.4^13^ was used with default settings to generate maximum-likelihood trees based on SNP alignment. As reference genomes, chromosome sequences of *E. coli* ST38 strain 144 (accession number NZ_CP023364.1), ST57 strain NCTC10444 (NZ_LR134092.1), ST117 strain 14EC020 (NZ_CP024138.1), ST350 strain NCTC9112 (NZ_LR134079.1), ST443 strain 2014C-3307 (NZ_CP027368.1), and ST2179 strain BR03-DEC (NZ_CP035321.1) were used. On the other hand, for ST2040 and ST3258, which had no complete genome assemblies available on Enterobase, we used as reference genomes the chromosome sequences of ST191 (closely related to ST2040) strain 1,500 (NZ_CP040269.1) and ST117 (closely related to ST3258) strain 14EC020 (NZ_CP024138.1). Finally, for FCC10, we used the closely related ST641 strain 1916D18 (NZ_CP046000.1). All reference genomes were chosen based on Enterobase search results. All genome assemblies used on phylogenetic analysis were screened for resistance genes and plasmid replicons using ABRicate v1.0.1 (see text footnote 7) with ResFinder and PlasmidFinder databases. iTOL v6^14^ was used to root the trees at midpoint and to annotate the trees with Enterobase and ABRicate data.

## 3 Results

A total of 14 *E. coli* isolates were recovered from 32 collected meat samples, mostly from chicken (12/14), including from both CN and AF production systems, followed by swine (2/14) samples obtained from CN alone (Supplementary Table S1). No *E. coli* strains were isolated from beef. Based on the sources of the meat samples, a higher frequency of *E. coli* isolates was obtained from chicken breast filet. A greater number of resistant *E. coli* strains were isolated from AF compared to the CN meat (6/9, and 6/23, respectively). Regardless of the storage method of meat samples, antimicrobial-resistant *E. coli* was mainly detected in FZ meat (6/8).

**Figure 1:**
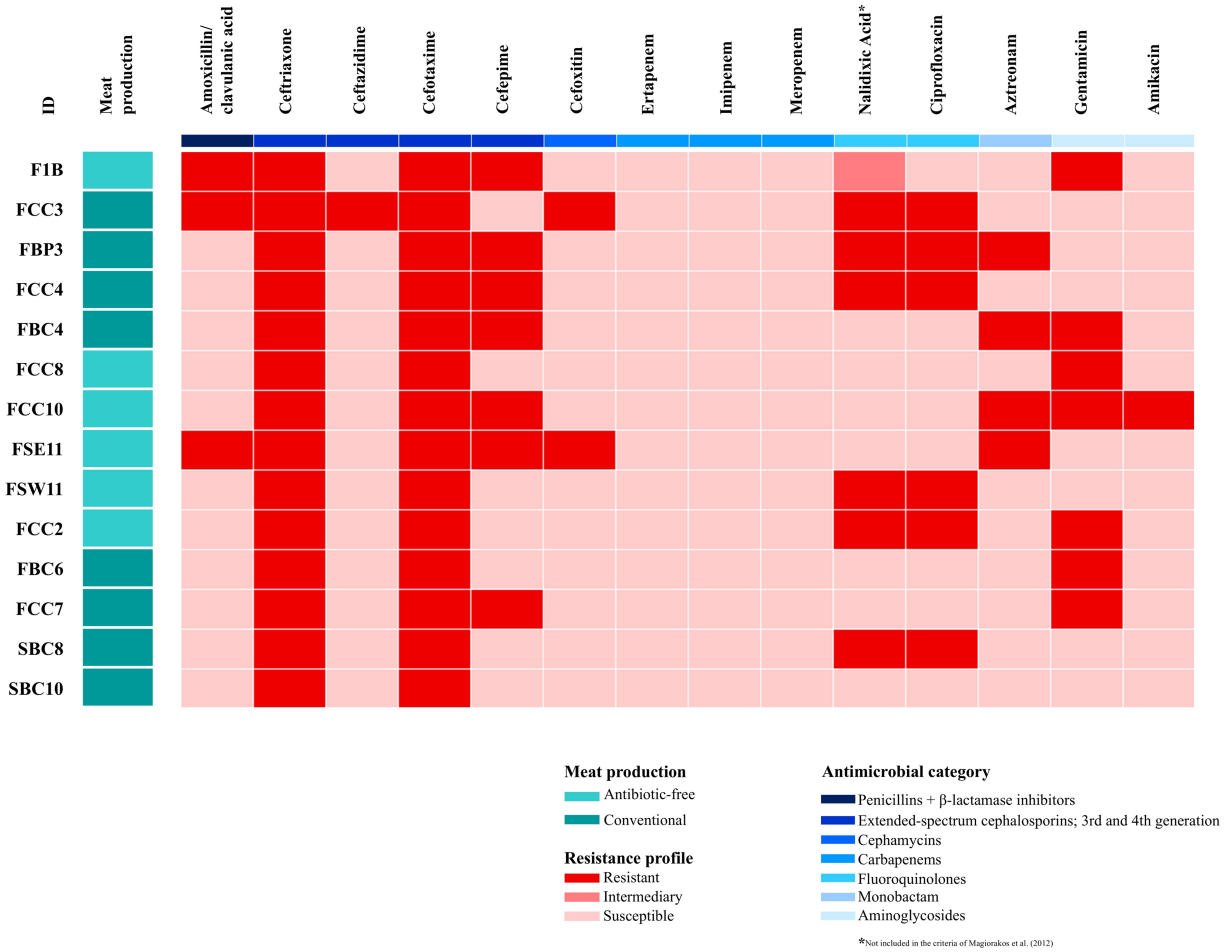
Heatmap showing the antimicrobial resistance profile of 14 *Escherichia coli* strains recovered from conventional and antibiotic-free retail meat in Brazil. Boxes highlighted in red indicate the antimicrobials for which the strains displayed resistance, while boxes in purple indicate susceptibility profiles. Aqua green color denotes antibiotic-free production systems, and dark green represents conventional systems.

Antimicrobial susceptibility testing of the 14 *E. coli* isolates is shown in Figure 1. The highest resistance frequency was found for ceftriaxone and cefotaxime (100% of the tested strains), followed by cefepime, nalidixic acid and gentamicin (50%). The seven *E. coli* strains (F1B, FCC3, FBP3, FBC4, FCC10, FSE11, and FCC2) were classified as MDR according to the code previously established by Magiorakos et al. (2012). The MDR strains were more prevalent in chicken meat from antibiotic-free production systems (4/6) compared to conventional systems (3/8) (Figure 1). Among the tested strains, these nine strain, F1B, FCC3, FBP3, FCC4, FBC4, FCC8, FCC10, FSE11, and FSW11, were selected for whole genome sequencing based on their susceptibility profile and ES*β*L phenotype (Figure 1). We chose only chicken meat strains because they represented most of the isolates and allowed for a more consistent comparison between antibiotic-free and conventional production systems within the same meat type.

Initial analysis of the sequenced strains was performed using an *in silico* MLST approach that revealed completely distinct sequence types (STs) among all strains (ST38, ST57, ST117, ST350, ST443, ST15579, ST2179, ST2040, and ST3258).

**Figure 2:**
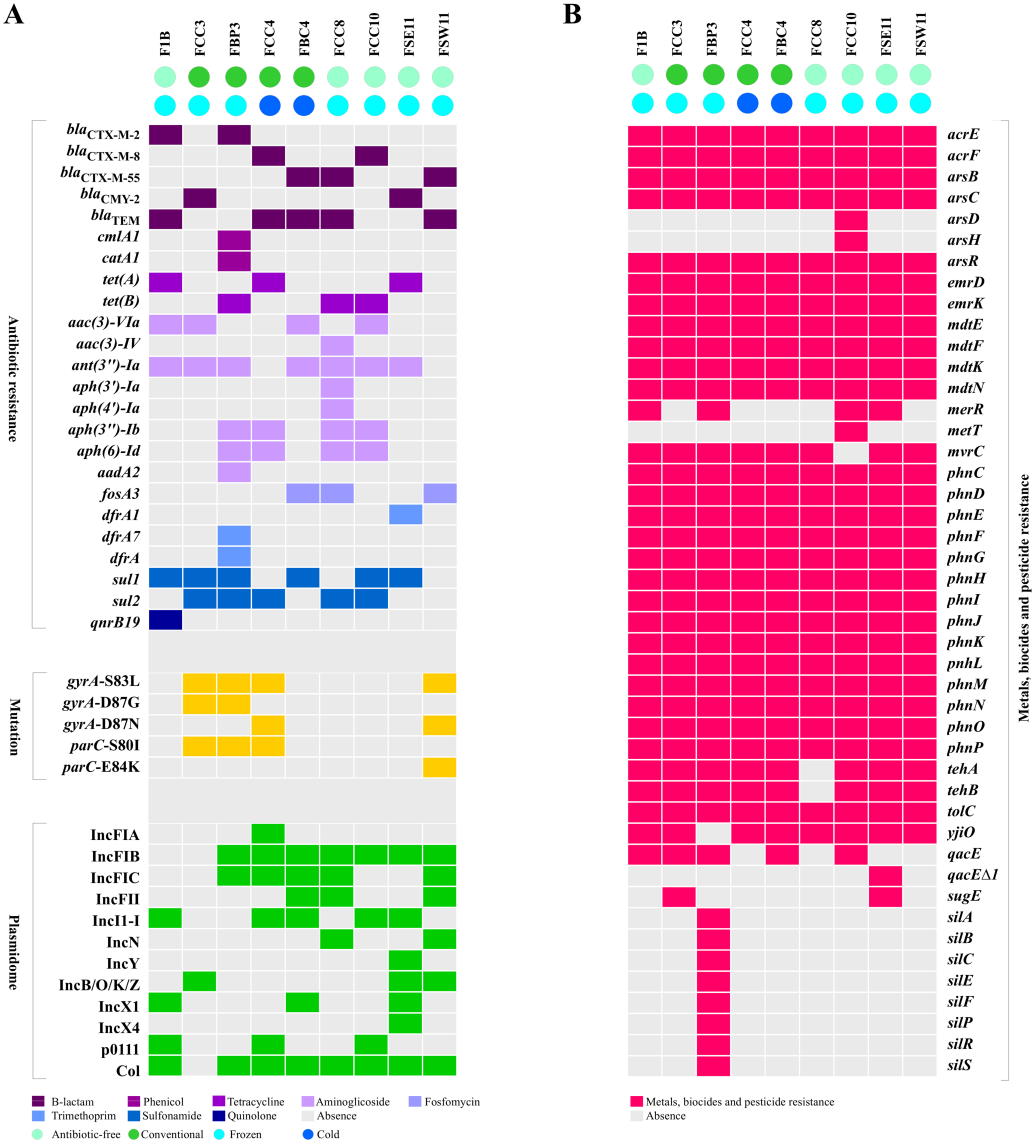
ESβL/pAmpC-producing *Escherichia coli* isolated from retail meat, considering the types of production (antibiotic-free or conventional) and storage (cold or frozen) system. **(A)** Antibiotic resistance, mutations and plasmidome diversity. **(B)** Metals, biocides and pesticide resistance. Highlighted boxes indicate the presence, while gray boxes indicate the absence of resistance determinants (to antimicrobials, metals, biocides, and pesticides), mutations, or plasmid incompatibility groups.

Overall, 63 antimicrobial resistance genes (ARGs) conferring resistance to eight antimicrobials categories, including β-lactams, phenicols, tetracyclines, aminoglycosides, fosfomycin, trimethoprim, quinolones, and sulfonamides were identified based on the WGS analysis (Figure 2A). Each genome of the nine strains harbored between three and 12 ARGs. The ß-lactam resistance genes *bla*_CMY-2_ or *bla*_CTX-M_ (*bla*_CTX-M-2_, *bla*_CTX-M-8_, or *bla*_CTX-M-55_ variants) were detected in all sequenced *E. coli* strains, regardless of whether from CN or AF production systems (Table 1). Among these, the *bla*_CTX-M-55_ gene variant was the most frequently found in *E. coli* strains, being identified in three strains (FBC4/CN, FCC8/AF, and FSEW11/AF).

Resistance to fluoroquinolones was mainly associated with chromosomal mutations on *gyrA* [S83L, D87N, and D87G] and *parC* [S80I and E84K] genes. Only the F1B/AF *E. coli* strain presented gene associated with plasmid mobilization (*qnrB19*).

The sequenced strains showed a broad resistome related to heavy metals, herbicide, and disinfectants resistance genes, ranging between 28 and 39 resistance genes in each strain (Figure 2B). Overall, all of them carried resistance genes to heavy metal (arsenic: *arsB, arsC,* and *arsR* genes), herbicide (glyphosate: *phnCDEFGHIJKLMNOP* genes), and disinfectants (*acrE, acrF, emrD, emrK, mdtE, mdtF, mdtK, mdtN*, and *tolC* genes).

Our results also identify 12 distinct plasmid replicon types among the 9 genomes analyzed, including IncFIA, IncFIB, IncFIC, IncFII, IncI1-I, IncN, IncY, IncB/O/K/Z, IncX1, IncX4, p0111, and Col (Figure 2A). The most prevalent plasmid replicons were Col (88.9%, 8/9) and IncFIB (77.8%, 7/9). Interestingly, the *E. coli* strains harboring the highest number of resistance genes, FBP3/ST38/CN (12 ARGs) and FCC8/ST3258/AF (11 ARGs), commonly exhibited the plasmids IncFIB, IncFIC, and Col. Additionally, FCC8 strain also possessed IncFII and IncN plasmids. Although the short-read methodology employed did not allow for the circularization of plasmids, using the mlplasmid tool, we detected plasmid-derived sequences carrying the *bla*_CTX-M_-type or *bla*_CMY-2_ genes in F1B, FCC3, FBP3, and FSE11 strains. As a limitation, the study did not include plasmid electrophoretic analysis.

**Figure 3:**
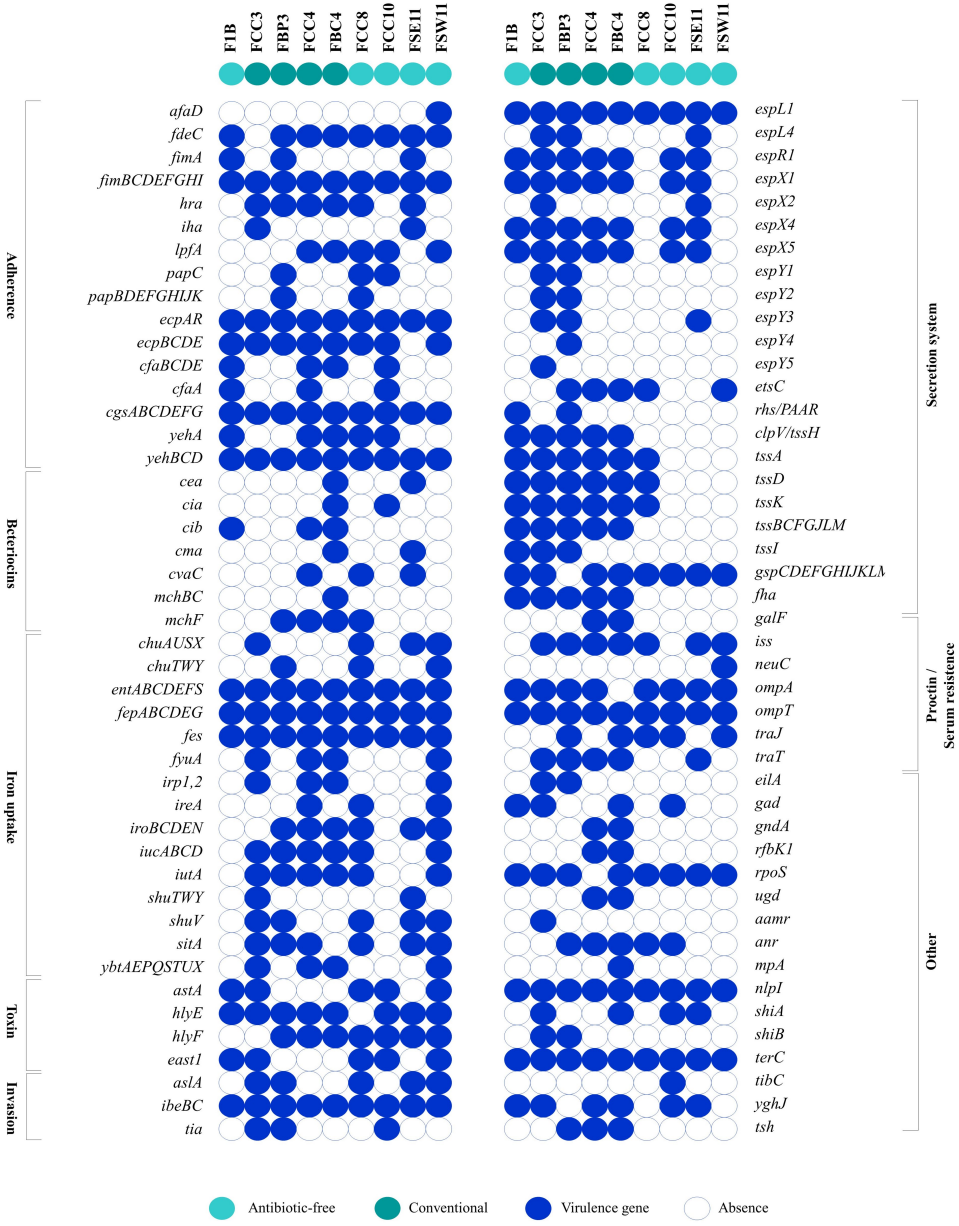
Comparative panel of virulence genes detected among antibiotic-free and conventional ESβL/pAmpC-producing *Escherichia coli* strains. The virulome scenario involves genes related to adherence, bacteriocins, iron uptake, toxins, invasion, secretion systems, protectins/serum resistance, and other factors of pathogenicity. Blue boxes represent the presence of virulence genes, while white boxes indicate their absence. Aqua green color denotes antibiotic-free production systems, and dark green represents conventional systems.

In total, 171 virulence genes representing different virulence pathogenicity mechanisms (adherence, bacteriocins, iron uptake, toxins, invasion, secretion systems, protectins/serum resistance, and other factors) were identified (Figure 3). The *E. coli* FBC4/ST2040 and FCC3/ST350 strains, from CN production system, harbored a higher number of virulence genes (124 and 120, respectively), followed by the FCC4/ST2179 (119 genes) and FBP3/ST38 (110 genes) strains. Despite this, those isolates from AF meat, FCC8/ST3258 and FSW11/ST117, also presented a broad virulome (104 and 100, respectively). All sequenced strains presented genes involved in processes of adherence (*fimBCDEFGHI, ecpAR, cgsABCDEFG,* and *yehBCD* genes), iron uptake (*entABCDEFS, fepABCDEG*, and *fes* genes), invasion (*ibeBC* genes), secretion system (*espL1* gene), proctin/ serum resistance (*ompT* gene), among others (*nlpI*, and *terC* genes). Detailed genomic information of virulome is shown in Supplementary Table S1.

Fimbriae and serotyping analysis, accessed by FimTyper and SerotypeFinder, respectively, showed that all strains were distinct, although strains FCC3/CN, FCC8/AF and FSW11/AF presented the same H antigen (H4) (Supplementary Table S1). The most frequent Clermont phylogroup was B1 type (3/9), followed by E type (2/9) (Supplementary Table S1).

**Figure 4:**
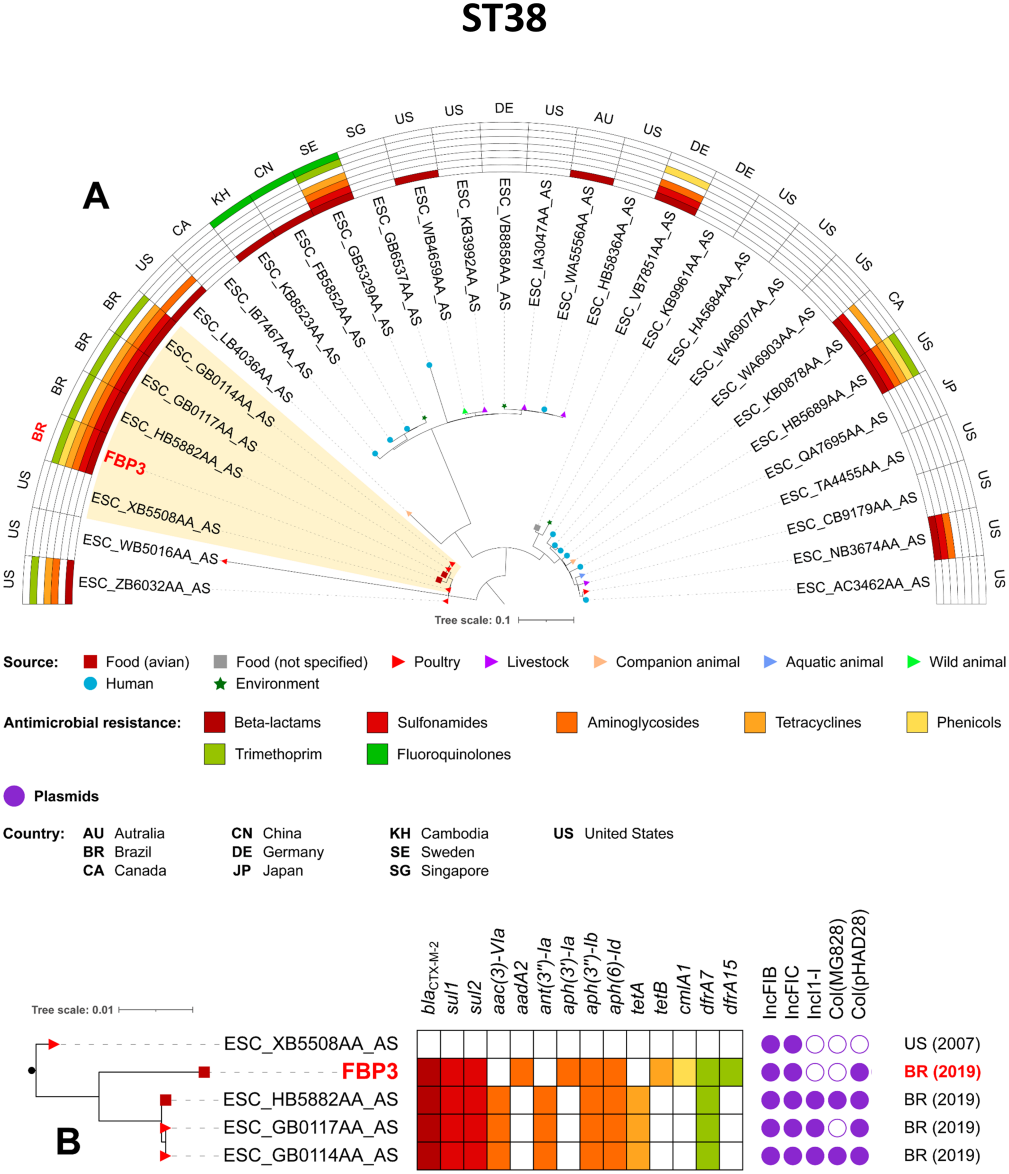
Phylogenomic analysis of FBP3 *Escherichia coli* strain belonging to ST38. In **A**, maximum-likelihood phylogenetic tree illustrating of 31 *Escherichia coli* ST38. Comparison of resistomes, isolation sources, and countries of origin of the strains. In **B**, zooming into the subtree comprising the clade A. The figure was generated with iTOL version 5.6.1 (https://itol.embl.de).

**Figure 5:**
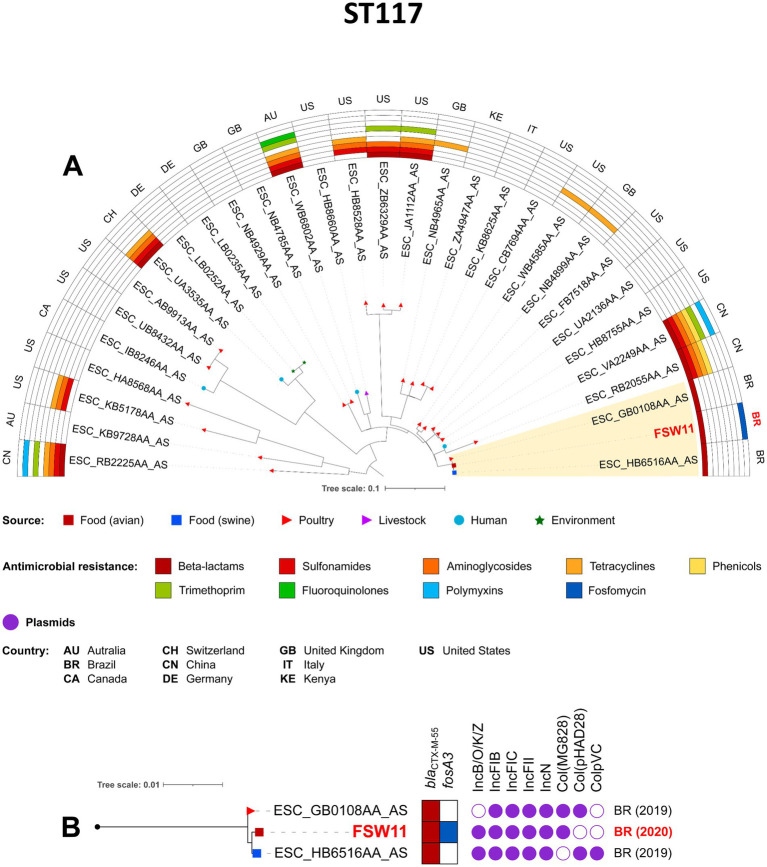
Phylogenomic analysis of FSW11 *Escherichia coli* strain belonging to ST117. In **A**, maximum-likelihood phylogenomic tree of 31 *E. coli* ST117. Comparison of resistomes, isolation sources, and countries of origin of the strains. In **B**, zooming into the subtree comprising the clade A. The figure was generated with iTOL version 5.6.1 (https://itol.embl.de).

The phylogenetic analysis was conducted based on sequence typing (ST), so only one isolate from our study (FBP3) was included in the analysis of the pandemic clone ST38, together with other related isolates. Notably, the FBP3/CN/FZ strain was closely related to chicken meat isolates from Brazil in 2019, and to poultry isolates from USA in 2007, with SNP differences ranging from 128 to 142, respectively (Figure 4; Supplementary Table S1). All Brazilian strains carried *bla*_CTX-M-2_, *sul1, sul2, aph(3″)-Ib, aph(6)-Id* and *drfA17* genes. The ST117 FSW11 (AF/FZ) strain showed close phylogenetic relatedness to other CTX-M-55-producing *E. coli* isolates from Brazil (2019), specifically those derived from swine meat (15 SNP differences) and poultry (17 SNP differences) (Figure 5; Supplementary Table S1). Additionally, the FBP3 and FSW11 strains were genomically linked (995–1843 SNPs) to the ST38 and ST117 lineages previously identified in environmental contamination in Europe (Figures 4, 5; Supplementary Table S1). From a plasmidome perspective, all ST117 strains carried the IncFIB, IncFIC, IncFII, and IncN plasmid groups (Figure 5). The presence of these plasmids in all strains suggests a high risk of antibiotic resistance spread, as these plasmid groups are known for efficient horizontal gene transfer and carriage of resistance genes (Liu et al., 2024; Felix et al., 2024; Yu et al., 2024).

In general, the F1B, FCC8, FBC4, FSE11 strains were nested within a food (avian), poultry and/or livestock clade (Supplementary Figure S1). On the other hand, the phylogenetic analysis of FCC4, FCC3, and FCC10 strains identified a clade comprising isolates from diverse sources, including avian food products, poultry, livestock, humans, and/or wild animals (Supplementary Figures S1B3,B5,B6). Furthermore, it was observed that some strains (FBP3, FCC4, and FBC4) share similar antibiotic resistance and plasmid content with their closest phylogenetic relatives, indicating the possible influence of horizontal gene transfer and environmental factors.

## 4 Discussion

Bidirectional transmission of antibiotic resistance is likely between humans, the food chain, and the environment. In this regard, industrial chicken farms represent an ideal environment for the spread of AMR bacteria, since the overuse of antimicrobials contributes to the selection of antibiotic-resistant bacteria in the gastrointestinal microbiota of animals, whereas the consumption of contaminated animal-source food, or environmental exposure can lead to the transmission of antimicrobial-resistant bacteria to humans and non-human hosts. In fact, antimicrobial resistance is regularly manifested in human clinical settings through food chain, with additional environmental spillovers.

This study prioritized the isolation and specific characterization of prevalent critical pathogens, following the FAO Action Plan and WHO’s global research guidelines on antimicrobial resistance (Keck et al., 2023; Bertagnolio et al., 2024). Therefore, as a limitation of this study, bacterial load was not measured before overnight enrichment, and virulence gene expression was not quantified. We report the occurrence of international clones of ESβL/pAmpC-positive *E. coli* isolated from both CN and AF retail meat sold at supermarkets across all regions of São Paulo, the most populous city in Latin America with over 11 million inhabitants (IBGE, 2025). This metropolitan area represents a diverse consumer population and a significant market for retail meat, making it a relevant setting for studying antimicrobial-resistant pathogens in food.

The dissemination of genes encoding ESβL in meat requires closer attention, particularly due to its association with the successful expansion of international high-risk clones (Fuga et al., 2022; Soncini et al., 2022). In this report, we highlight the occurrence of CTX-M-producing *E. coli* strains belonging to diverse lineages, including ST38, ST57, ST117, ST350, ST443, ST15579, ST2179, ST2040, and ST3258. While CTX-M-producing clones belonging to ST350 have already been widely reported in chicken samples from southwest China (Li et al., 2022), our phylogeographical analysis further confirmed the presence of ST350 clone in poultry samples from the USA, as well as its clonal relationship with the *E. coli* FCC3 strain identified in our study. Strikingly, *E. coli* ST350 has been reported since 1979 (accession number AASASW000000000.1), highlighting a long-standing circulation and persistence.

The *E. coli* ST3258 present in organic broiler farm has been detected in Netherlands (van Hoek et al., 2018) and ST2040 and has been mainly reported to harboring the *bla*_CMY-2_ gene (Castellanos et al., 2017; Che et al., 2023). The other STs (ST57, ST443, and ST2179) have been reported in Brazil associated with *bla*_CTX-M_-type or *bla*_CMY-2_ genes, being recovered from different hosts and sources (Fuga et al., 2022; Leigue et al., 2015; Palmeira et al., 2018; Dos Anjos Adur et al., 2022). To our knowledge, this is the first report of an *E. coli* ST15579 strain. The presence of closely related ST15579-FCC10, ST2179-FCC4, and ST350-FCC3 strains across avian, livestock, human, and/or wild animal hosts underscores potential transmission pathways at the human-animal-environment interface, emphasizing the importance of a One Health approach to antimicrobial resistance surveillance.

Worryingly, we identified the presence of high-risk lineages ST38 and ST117 in chicken meat. These *E. coli* clones have been increasingly identified in various sources including food, animals, humans, and the environment, emphasizing their high versatility at the One Health interface (Fuga et al., 2022; Soncini et al., 2022; Berg et al., 2017; Mo et al., 2023). Critically, these strains were recovered from chicken meat of AF production systems, which might suggest contamination during the food chain process or storage. In this regard, the AF meat production has been proposed to minimize the use of antimicrobials as prophylactic agents and growth promoters (World Health Organization, 2017; Food and Agriculture Organization of the United Nations, 2019; Tang et al., 2019; Murray et al., 2021). Indeed, there is global pressure to reduce the use of antimicrobials in chicken and swine production, as it raises concerns about the development of antimicrobial resistance (AMR) and its potential impact on public health (World Health Organization, 2017; Food and Agriculture Organization of the United Nations, 2019).

In the last years, the pandemic antimicrobial-resistant *E. coli* clone ST38 has been reported as a common environmental bacterial contaminant disseminated through hospital sewage in Norway, municipal wastewater in Croatia, wastewater treatment plants and rivers in China and Tunisia, hospital and community wastewater in Czech Republic, surface waters and sewage in Ireland, estuaries in Lebanon, and river water in Algeria (Grevskott et al., 2024; Puljko et al., 2024; Li et al., 2023; Davidova-Gerzova et al., 2023; Hooban et al., 2021; Hassen et al., 2021; Diab et al., 2018; Seni et al., 2018; Tafoukt et al., 2017; Oikarainen et al., 2019); serving, indeed, as an environmental sentinel for AMR. Strikingly, *E. coli* ST38, have been found to intersect variably across the human-animal-environment interface in Switzerland and Brazil (Fuga et al., 2022; Müller et al., 2016). On the other hand, *E. coli* ST117, one of the extended-spectrum *β*-lactamase (ESβL)-producing clone that we isolated in this study, has been previously found in chicken meat in Spain, imported Brazilian poultry meat, and human extraintestinal disease, presenting a risk to humans ingesting poultry products (Martínez-Álvarez et al., 2025; Saidenberg et al., 2024; da Silva et al., 2022; Casella et al., 2018).

Remarkably, our study revealed a higher number of antimicrobial-resistant *E. coli* strains recovered from meat sourced from AF farming systems.

The presence of antibiotic-resistant bacteria in antibiotic-free meat may seem contradictory and does not always mean antibiotics were used in poultry production (Davis et al., 2018; Rawat et al., 2024). In this regard, several factors could explain this phenomenon, including: (i) imported chicks or feed additives containing antibiotic-resistant bacteria; (ii) horizontal transmission of antibiotic-resistant bacteria from other animals, farm workers, or contaminated surfaces; (iii) soil, water, and/or feed contaminated with resistant bacteria, from neighboring farms or past practices (as resistant bacteria are known to persist in the environment and can colonize animals raised without antibiotics); and, (iv) unintentional cross-contamination during processing or transport (Adegbeye et al., 2024; Coppola et al., 2022; Tian et al., 2021; Sun et al., 2024; Thanner et al., 2016; Argudín et al., 2017; Park et al., 2017; De Cesare et al., 2022; Millman et al., 2013). In slaughterhouses that process animals from multiple sources, cross-contamination can occur if facilities are not properly cleaned, where tools, conveyor belts for poultry processing plant, and handlers can spread bacteria from one carcass to another (Warriner et al., 2002; Bergšpica et al., 2020; Park et al., 2017; De Cesare et al., 2022). While poor hygiene, inadequate disinfection, or substandard biosecurity practices on antibiotic-free farms can allow resistant bacteria to thrive or spread, resistance genes can persist in microbial communities for extended periods (Jaleta et al., 2024; Johnsen et al., 2009).

On the other hand, the meaning of antibiotic-free products can cause some confusion, mainly because there is no official or international accepted definition of what it is and how to classify different types of antibiotic-free products. Additionally, complications come with anticoccidials for prevention of coccidiosis in poultry, since in some countries they are classified as antimicrobials and as such they must be withdrawn from antibiotic-free production. In some countries, labels on retail poultry have been a source of misunderstanding to the consumer. Meat from chickens raised with sub-therapeutic doses of antibiotics may still contain labels claiming, “all natural” or “free range,” which imply a healthier product even though both statements are silent on antibiotics use. For retail poultry meat, the label “organic” [defined and certified by the United States Department of Agriculture (USDA)], implies not using antibiotics or hormones in poultry livestock after the first 24 h of life. Thus, injecting antibiotics into eggs or administering antibiotics to one-day-old chicks are practices that are often performed and do not violate the USDA organic standard (Sanchez et al., 2020). Moreover, various forms of “no antibiotic” labels have been used, such as raised without antibiotics (RWA), no antibiotics administered, no added antibiotics, or raised antibiotic free, which describe meat from chicken that has not been administered antibiotics during production. However, the “no antibiotics ever (NAE)” label seem to be slightly stricter than the others, as it also restricts the antibiotic use in the egg (Sanchez et al., 2020; Singer et al., 2019). Most likely, the antibiotic-free label should guarantee that the meat being sold does not carry detectable levels of antibiotics.

In brief, retail poultry products have been known sources of antibiotic-resistant *E. coli*, and although consumers have a range of choices for poultry meat, including conventional, organic, and antibiotic-free designations, which are used to indicate differences in quality and safety, the frequency of contamination with antibiotic-resistant *E. coli* in food sold in these categories is unknown.

Although a limitation of our study is that we did not determine the source of contamination of commercialized chicken meat, there is no doubt that the occurrence of ESβL-producing *E. coli* in food sold for human consumption should not occur. In this respect, the food chain has globally been recognized as a reservoir and critical pathway for the development and dissemination of AMR, involving farming, processing, transportation, distribution, storage, retail and consumption (Founou et al., 2021; Choy et al., 2024; De Cesare et al., 2022); whereas the dissemination of *bla*_CTX-M_-type ESβL genes in human health is one of the main problems related to broad-spectrum cephalosporin resistance, particularly when associated with the spread of successful pandemic clones (Chong et al., 2018). Therefore, the presence of CTX-M-producing *E. coli* in retail chicken poses risk to human health, and studies investigating human acquisition through food consumption are necessary. Although it has been suggested that the consumption of chicken meat could be related to the acquisition of ESβL-producing *E. coli* and urinary tract infections, in most cases, human infections with ESβL-/pAmpC-producing *E. coli* are preceded by asymptomatic carriage (Manges et al., 2007; Isendahl et al., 2019; Plaza-Rodríguez et al., 2021; Dantas et al., 2025).

Further studies must also be directed to understand the evolutionary changes of CTX-M-positive *E. coli* in poultry meat, evaluate biofilm-forming capacity on the food chain, investigate interactions with food microbiota, develop quantitative microbial risk assessment models to estimate the risk of human and animal exposure, and expand surveillance. On the other hand, to assess the presumptive food safety and microbiological quality of foods, in addition to estimating bacterial numbers (i.e., total coliform and *E. coli* counts) as indicators of unfavorable hygienic conditions and fecal contamination in food, the absence of WHO critical priority Enterobacterales (resistant to broad-spectrum cephalosporins and/or carbapenems) in chicken meat should be used as a microbiological standard. On the other hand, the occurrence of CTX-M-positive *E. coli* in food has significant environmental and public health implications, including: (i) potential for horizontal gene transfer; (ii) long-term persistence in food waste; (iii) colonization of companion animals and/or humans, via contaminated food, creating new reservoirs for AMR; and, (iv) risk of community-acquired infections, especially among immunocompromised individuals.

Finally, widespread dissemination of ESβL (CTX-M)-positive *E. coli* has been favored by globalization of food trade, and this could be contributing to the successful dissemination of international clones, reaching parts of the world where they were not previously present (Dhanji et al., 2010; Kawamura et al., 2014; Egervärn et al., 2014; Nahar et al., 2018; Kim et al., 2018; Eibach et al., 2018; Campos et al., 2018; Kelbert et al., 2025). Given the global dynamics of ESβL transmission, a multisectoral and multidisciplinary approach is critical to the success of the global action plan on AMR.

Tetracyclines, *β*-lactams (penicillins), aminoglycosides, quinolone and sulfonamides are among the most widely used classes of antimicrobials in food-producing animals worldwide (Centner, 2016; Caneschi et al., 2023). Interestingly, in this study, all *E. coli* strains carried genes conferring resistance to β-lactams, and the majority (8/9, 88.9%) exhibited one or more genes conferring resistance to aminoglycosides and sulfonamides. Additionally, several strains also harbored mechanisms conferring resistance to tetracycline and quinolones. In line with our findings, some studies have indicated that there is no significant disparity between meat from AF and CN production system related to AMR genes (Ferri et al., 2023; Farooq et al., 2022; Rawat et al., 2024).

The plasmids IncFIB and Col were the most frequently found in the strains analyzed, suggesting a high risk of antibiotic resistance spread, as these plasmid groups are known for efficient horizontal gene transfer and carriage of resistance genes (Liu et al., 2024; Felix et al., 2024).

Regarding the virulome, its context has been studied among lineages belonging to specific phylogroups of *E. coli* (Beghain et al., 2018). In this study, the FBC4 strain, belonging to phylogroup A* (with potential mutation), was the one that presented the largest set of virulence genes. Despite this, strains of phylogroup A are normally associated with commensal lineages (Mosquito et al., 2015).

Another interesting point is the presence of critical-priority *E. coli* in FZ meat. The ability of these bacteria to survive in low-temperature stress has already been demonstrated (Parvin et al., 2020), underscoring the significance of processors adopting and adhering to good slaughtering and processing practices.

## 5 Conclusion

In summary, we report the detection of global WHO critical priority clones of CTX-M-type/pAmpC-producing *E. coli* in commercially available FS and FZ chicken meat from both AF and CV production systems in Brazil, which is considered a major global chicken meat exporter. We highlight that meat could serve as potential reservoirs and vectors of medically important antimicrobial-resistant bacteria, posing a significant threat to consumers. Merely discontinuing the use of antimicrobials in food-producing animals without addressing other factors may not fully resolve the issue of AMR in the meat industry and food safety. Our findings raise questions about the efficacy of current agricultural practices, antimicrobial usage in animal husbandry, and potential routes of contamination during meat processing and distribution. Addressing these issues is crucial and requires collaborative efforts among stakeholders in the food industry, veterinary and medical sectors, and governmental agencies to implement strategies aimed at reducing the prevalence of these critical-priority bacteria in meat. Hence, it is imperative to adopt multi-faceted approaches across the meat production chain, including better farm practices and biosecurity, responsible use of antimicrobials, strict hygiene practices, proper storage and transportation, effective regulatory measures, and education – supplemented by continuous surveillance of AMR in these products, to effectively mitigate contamination routes.

## Data Availability

This Whole Genome Shotgun project has been deposited at DDBJ/ENA/GenBank under the accessions JAQQRG000000000 (F1B), JABEPS000000000 (FCC3), JABEPT000000000 (FBP3), JABEPU000000000 (FCC4), JAQQRI000000000 (FBC4), JAQQYK000000000 (FCC8), JAQQYJ000000000 (FCC10), JAQQYM000000000 (FSE11), and JAQQYL000000000 (FSW11). Additionally, genomic data are available at the OneBR platform under the number IDs ONE135 (F1B), ONE136 (FCC3), ONE137 (FBP3), ONE138 (FCC4), ONE139 (FBC4), ONE140 (FCC8), ONE141 (FCC10), ONE142 (FSE11), and ONE143 (FSW11) (http://onehealthbr.com/).

## Footnotes

^1^ https://github.com/FelixKrueger/TrimGalore

^2^ https://github.com/rrwick/Unicycler

^3^ http://www.ncbi.nlm.nih.gov/genome/annotation_prok/

^4^ https://pubmlst.org/

^5^ https://enterobase.warwick.ac.uk

^6^ https://www.genomicepidemiology.org

^7^ https://github.com/tseemann/abricate

^8^ https://github.com/haruosuz/vfdb

^9^ http://bacmet.biomedicine.gu.se/

^10^ http://clermontyping.iame-research.center

^11^ https://sarredondo.shinyapps.io/mlplasmids/

^12^ https://github.com/ParBLiSS/FastANI

^13^ https://cge.food.dtu.dk/services/CSIPhylogeny/

^14^ https://itol.embl.de

## Supplementary material

SUPPLEMENTARY FIGURE S1 Phylogenetic analysis. In **A1–A7**, maximum-likelihood phylogenetic tree of F1B (ST443), FCC8 (ST3258), FCC4 (ST2179), FBC4 (ST2040), FCC3 (ST350), FCC10 (ST15579), and FSE11 (ST57) *Escherichia coli* strains. Comparison of resistomes, isolation sources, and countries of origin of the strains. In **B1–B7**, zooming into the subtree comprising sequenced strains of *Escherichia coli* in this study. The figure was generated with iTOL version 5.6.1 (https://itol.embl.de).

SUPPLEMENTARY TABLE S1 Epidemiological characteristics, genetic repertoire, and SNP matrix of ESβL/pAmpC-producing *Escherichia coli* isolated from conventional and antibiotic-free retail meat in Brazil.

## References

[ref1] Adegbeye M. J. Adetuyi B. O. Igirigi A. I. Adisa A. Palangi V. Aiyedun S.. (2024). Comprehensive insights into antibiotic residues in livestock products: distribution, factors, challenges, opportunities, and implications for food safety and public health. Food Control 163:110545. doi: 10.1016/j.foodcont.2024.110545

[ref2] Alegría Á. Arias-Temprano M. Fernández-Natal I. Rodríguez-Calleja J. M. García-López M. L. Santos J. A. (2020). Molecular diversity of ESΒL-producing *Escherichia coli* from foods of animal origin and human patients. Int. J. Environ. Res. Public Health 17:1312. doi: 10.3390/ijerph17041312, PMID: 32085569 PMC7068493

[ref3] Antimicrobial Resistance Collaborators (2022). Global burden of bacterial antimicrobial resistance in 2019: a systematic analysis. Lancet 399, 629–655. doi: 10.1016/S0140-6736(21)02724-0, PMID: 35065702 PMC8841637

[ref4] Argudín M. A. Deplano A. Meghraoui A. Dodémont M. Heinrichs A. Denis O.. (2017). Bacteria from animals as a Pool of antimicrobial resistance genes. Antibiotics 6:12. doi: 10.3390/antibiotics6020012, PMID: 28587316 PMC5485445

[ref5] Aslam B. Khurshid M. Arshad M. I. Muzammil S. Rasool M. Yasmeen N.. (2021). Antibiotic resistance: one health one world outlook. Front. Cell. Infect. Microbiol. 11:771510. doi: 10.3389/fcimb.2021.771510, PMID: 34900756 PMC8656695

[ref6] Beghain J. Bridier-Nahmias A. Le Nagard H. Denamur E. Clermont O. (2018). Clermontyping: an easy-to-use and accurate in silico method for *Escherichia* genus strain phylotyping. Microb. Genom. 4:e000192. doi: 10.1099/mgen.0.000192, PMID: 29916797 PMC6113867

[ref7] Berg E. S. Wester A. L. Ahrenfeldt J. Mo S. S. Slettemeås J. S. Steinbakk M.. (2017). Norwegian patients and retail chicken meat share cephalosporin-resistant *Escherichia coli* and Inc K/*bla*_CMY-2_ resistance plasmids. Clin. Microbiol. Infect. 23, 407.e9–407.e15. doi: 10.1016/j.cmi.2016.12.035, PMID: 28082191

[ref8] Bergšpica I. Kaprou G. Alexa E. A. Prieto M. Alvarez-Ordóñez A. (2020). Extended Spectrum β-lactamase (ESBL) producing *Escherichia coli* in pigs and pork meat in the European Union. Antibiotics 9:678. doi: 10.3390/antibiotics9100678, PMID: 33036406 PMC7600538

[ref9] Bertagnolio S. Dobreva Z. Centner C. M. Olaru I. D. Donà D. Burzo S.. (2024). WHO global research priorities for antimicrobial resistance in human health. Lancet Microbe 5:100902. doi: 10.1016/S2666-5247(24)00134-4, PMID: 39146948 PMC11543637

[ref10] Campos J. Mourão J. Silveira L. Saraiva M. Correia C. B. Maçãs A. P.. (2018). Imported poultry meat as a source of extended-spectrum cephalosporin-resistant CMY-2-producing *Salmonella* Heidelberg and *Salmonella* Minnesota in the European Union, 2014-2015. Int. J. Antimicrob. Agents 51, 151–154. doi: 10.1016/j.ijantimicag.2017.09.006, PMID: 28919197

[ref11] Caneschi A. Bardhi A. Barbarossa A. Zaghini A. (2023). The use of antibiotics and antimicrobial resistance in veterinary medicine, a complex phenomenon: a narrative review. Antibiotics 12:487. doi: 10.3390/antibiotics12030487, PMID: 36978354 PMC10044628

[ref12] Casella T. Haenni M. Madela N. K. Andrade L. K. Pradela L. K. Andrade L. N.. (2018). Extended-spectrum cephalosporin-resistant *Escherichia coli* isolated from chickens and chicken meat in Brazil is associated with rare and complex resistance plasmids and pandemic ST lineages. J. Antimicrob. Chemother. 73, 3293–3297. doi: 10.1093/jac/dky335, PMID: 30184138

[ref13] Castellanos L. R. Donado-Godoy P. León M. Clavijo V. Arevalo A. Bernal J. F.. (2017). High heterogeneity of *Escherichia coli* sequence types harbouring ESΒL/amp C genes on IncI1 plasmids in the Colombian poultry chain. PLoS One 12:e0170777. doi: 10.1371/journal.pone.0170777, PMID: 28125687 PMC5268450

[ref14] Centner T. J. (2016). Efforts to slacken antibiotic resistance: labeling meat products from animals raised without antibiotics in the United States. Sci. Total Environ. 563-564, 1088–1094. doi: 10.1016/j.scitotenv.2016.05.082, PMID: 27236477

[ref15] Che M. Birk T. Hansen L. T. (2023). Prevalence and transmission of extended-Spectrum cephalosporin (ESC) resistance genes in *Escherichia coli* isolated from poultry production systems and slaughterhouses in Denmark. Antibiotics 12:1602. doi: 10.3390/antibiotics12111602, PMID: 37998804 PMC10668726

[ref16] Chong Y. Shimoda S. Shimono N. (2018). Current epidemiology, genetic evolution and clinical impact of extended-spectrum β-lactamase-producing *Escherichia coli* and *Klebsiella pneumoniae*. Infect. Genet. Evol. 61, 185–188. doi: 10.1016/j.meegid.2018.04.005, PMID: 29626676

[ref17] Choy S. K. Neumann E.-M. Romero-Barrios P. Tamber S. (2024). Contribution of food to the human health burden of antimicrobial resistance. Foodborne Pathog. Dis. 21, 71–82. doi: 10.1089/fpd.2023.0099, PMID: 38099924 PMC10877391

[ref18] Clinical and Laboratory Standards Institute (2024). Performance standards for antimicrobial susceptibility testing. Thirty-first informational supplement M100-S34. Wayne, PA: CLSI.

[ref19] Coppola N. Cordeiro N. F. Trenchi G. Esposito F. Fuga B. Fuentes-Castillo D.. (2022). Imported one-day-old chicks as Trojan horses for multidrug-resistant priority pathogens harboring *mcr-9, rmtG*, and extended-Spectrum β-lactamase genes. Appl. Environ. Microbiol. 88:e0167521. doi: 10.1128/AEM.01675-21, PMID: 34731047 PMC8788672

[ref20] Da Silva C. R. do Valle B. M. Gozi K. S. Fontana H. Nogueira M. C. L. Lincopan N.. (2022). Genomic analysis of *Escherichia coli* circulating in the Brazilian poultry sector. Braz. J. Microbiol. 53, 2121–2131. doi: 10.1007/s42770-022-00799-x 35864380 PMC9679118

[ref21] Dantas K. Melocco G. Esposito F. Fontana H. Cardoso B. Lincopan N. (2025). Emergent *Escherichia coli* of the highly virulent B2-ST1193 clone producing KPC-2 carbapenemase in ready-to-eat vegetables. J. Glob. Antimicrob. Resist. 41, 105–110. doi: 10.1016/j.jgar.2024.11.020, PMID: 39674367 PMC11888992

[ref22] Davidova-Gerzova L. Lausova J. Sukkar I. Nesporova K. Nechutna L. Vlkova K.. (2023). Hospital and community wastewater as a source of multidrug-resistant ESBL-producing *Escherichia coli*. Front. Cell. Infect. Microbiol. 15:1184081. doi: 10.3389/fcimb.2023.1184081 PMC10225658 37256105

[ref23] Davis G. S. Waits K. Nordstrom L. Grande H. Weaver B. Papp K.. (2018). Antibiotic-resistant *Escherichia coli* from retail poultry meat with different antibiotic use claims. BMC Microbiol. 18:174. doi: 10.1186/s12866-018-1322-5, PMID: 30390618 PMC6215666

[ref24] De Cesare A. Oliveri C. Lucchi A. Savini F. Manfreda G. Sala C. (2022). Pilot study on poultry meat from antibiotic free and conventional farms: can metagenomics detect any difference? Food Secur. 11:249. doi: 10.3390/foods11030249, PMID: 35159402 PMC8834493

[ref25] Dhanji H. Murphy N. M. Doumith M. Durmus S. Lee S. S. Hope R.. (2010). Cephalosporin resistance mechanisms in *Escherichia coli* isolated from raw chicken imported into the UK. J. Antimicrob. Chemother. 65, 2534–2537. doi: 10.1093/jac/dkq376, PMID: 20889530

[ref26] Diab M. Hamze M. Bonnet R. Saras E. Madec J. Y. Haenni M. (2018). Extended-spectrum beta-lactamase (ESBL)- and carbapenemase-producing Enterobacteriaceae in water sources in Lebanon. Vet. Microbiol. 217, 97–103. doi: 10.1016/j.vetmic.2018.03.007, PMID: 29615264

[ref27] Dos Anjos Adur M. Châtre P. Métayer V. Drapeau A. Pillonetto M. Penkal M. L.. (2022). *Escherichia coli* ST224 and Inc F/*bla*_CTX-M-55_ plasmids drive resistance to extended-spectrum cephalosporins in poultry flocks in Parana, Brazil. Int J Food Microbiol 380:109885. doi: 10.1016/j.ijfoodmicro.2022.109885, PMID: 36057242

[ref28] Drieux L. Brossier F. Sougakoff W. Jarlier V. (2008). Phenotypic detection of extended-spectrum beta-lactamase production in Enterobacteriaceae: review and bench guide. Clin. Microbiol. Infect. 14, 90–103. doi: 10.1111/j.1469-0691.2007.01846.x, PMID: 18154532

[ref29] Egervärn M. Börjesson S. Byfors S. Finn M. Kaipe C. Englund S.. (2014). *Escherichia coli* with extended-spectrum beta-lactamases or transferable AmpC beta-lactamases and Salmonella on meat imported into Sweden. Int. J. Food Microbiol. 171, 8–14. doi: 10.1016/j.ijfoodmicro.2013.11.005, PMID: 24296257

[ref30] Eibach D. Dekker D. Gyau Boahen K. Wiafe Akenten C. Sarpong N. Belmar Campos C.. (2018). Extended-spectrum beta-lactamase-producing *Escherichia coli* and *Klebsiella pneumoniae* in local and imported poultry meat in Ghana. Vet. Microbiol. 217, 7–12. doi: 10.1016/j.vetmic.2018.02.023, PMID: 29615260

[ref31] Farooq M. Smoglica C. Ruffini F. Soldati L. Marsilio F. Di Francesco C. E. (2022). Antibiotic resistance genes occurrence in conventional and antibiotic-free poultry farming, Italy. Animals 12:2310. doi: 10.3390/ani12182310, PMID: 36139170 PMC9495165

[ref32] Felix M. A. Sopovski D. Commichaux S. Yoskowitz N. Aljahdali N. H. Grim C. J.. (2024). Genetic relatedness and virulence potential of *Salmonella* Schwarzengrund strains with or without an IncFIB-IncFIC (FII) fusion plasmid isolated from food and clinical sources. Front. Microbiol. 15:1397068. doi: 10.3389/fmicb.2024.1397068, PMID: 38827152 PMC11143878

[ref33] Ferri G. Buonavoglia A. Farooq M. Festino A. R. Ruffini F. Paludi D.. (2023). Antibiotic resistance in Italian poultry meat production chain: a one-health perspective comparing antibiotic free and conventional systems from the farming to the slaughterhouse. Front. Food Sci. Technol. 3:1168896. doi: 10.3389/frfst.2023.1168896

[ref34] Food and Agriculture Organization of the United Nations. (2019). Prudent and efficient use of antimicrobials in pigs and poultry. Available online at: https://www.fao.org/documents/card/fr/c/CA6729EN (Accessed January 15, 2025).

[ref35] Foster-Nyarko E. Pallen M. J. (2022). The microbial ecology of *Escherichia coli* in the vertebrate gut. FEMS Microbiol. Rev. 46:fuac008. doi: 10.1093/femsre/fuac008, PMID: 35134909 PMC9075585

[ref36] Founou L. L. Founou R. C. Essack S. Y. (2021). Antimicrobial resistance in the farm-to-plate continuum: more than a food safety issue. Future Sci. OA 7:FSO692. doi: 10.2144/fsoa-2020-0189, PMID: 34046194 PMC8147750

[ref37] Fuga B. Sellera F. P. Cerdeira L. Esposito F. Cardoso B. Fontana H.. (2022). WHO critical priority *Escherichia coli* as one health challenge for a post-pandemic scenario: genomic surveillance and analysis of current trends in Brazil. Microbiol. Spectr. 10:e0125621. doi: 10.1128/spectrum.01256-21, PMID: 35234515 PMC8941879

[ref38] Grevskott D. H. Radisic V. Salvà-Serra F. Moore E. R. B. Akervold K. S. Victor M. P.. (2024). Emergence and dissemination of epidemic-causing OXA-244 carbapenemase-producing *Escherichia coli* ST38 through hospital sewage in Norway, 2020-2022. J. Hosp. Infect. 145, 165–173. doi: 10.1016/j.jhin.2023.12.020, PMID: 38286237

[ref39] Haque M. H. Sarker S. Islam M. S. Islam M. A. Karim M. R. Kayesh M. E. H.. (2020). Sustainable antibiotic-free broiler meat production: current trends, challenges, and possibilities in a developing country perspective. Biology 9:411. doi: 10.3390/biology9110411, PMID: 33238534 PMC7700346

[ref40] Hassen B. Abbassi M. S. Ruiz-Ripa L. Mama O. M. Ibrahim C. Benlabidi S.. (2021). Genetic characterization of extended-spectrum β-lactamase-producing Enterobacteriaceae from a biological industrial wastewater treatment plant in Tunisia with detection of the colistin-resistance *mcr-1* gene. FEMS Microbiol. Ecol. 97:fiaa231. doi: 10.1093/femsec/fiaa231, PMID: 33202005

[ref41] Hooban B. Fitzhenry K. Cahill N. Joyce A. O' Connor L. Bray J. E.. (2021). A point prevalence survey of antibiotic resistance in the Irish environment, 2018-2019. Environ. Int. 152:106466. doi: 10.1016/j.envint.2021.106466, PMID: 33706038

[ref42] IBGE. (2025) Brazilian institute of geography and statistics. Available online at: https://cidades.ibge.gov.br/brasil/sp/sao-paulo/panorama (Accessed May 04, 2025).

[ref43] Isendahl J. Giske C. G. Hammar U. Sparen P. Tegmark Wisell K. Ternhag A.. (2019). Temporal dynamics and risk factors for bloodstream infection with extended-spectrum β-lactamase-producing bacteria in previously-colonized individuals: national population-based cohort study. Clin. Infect. Dis. 68, 641–649. doi: 10.1093/cid/ciy539, PMID: 29961883

[ref44] Jacob M. E. Keelara S. Aidara-Kane A. Matheu Alvarez J. R. Fedorka-Cray P. J. (2020). Optimizing a screening protocol for potential extended-spectrum β-lactamase *Escherichia coli* on MacConkey agar for use in a global surveillance program. J. Clin. Microbiol. 58:e01039-19. doi: 10.1128/JCM.01039-19, PMID: 32434784 PMC7448649

[ref45] Jaleta M. Junker V. Kolte B. Börger M. Werner D. Dolsdorf C.. (2024). Improvements of weaned pigs barn hygiene to reduce the spread of antimicrobial resistance. Front. Microbiol. 14:1393923. doi: 10.3389/fmicb.2024.1393923 PMC11135127 38812683

[ref46] Jarlier V. Nicolas M. H. Fournier G. Philippon A. (1998). Extended broad-spectrum beta-lactamases conferring transferable resistance to newer beta-lactam agents in Enterobacteriaceae: hospital prevalence and susceptibility patterns. Rev. Infect. Dis. 10, 867–878. doi: 10.1093/clinids/10.4.867 3263690

[ref47] Jesudason T. (2023). A new research agenda to combat antimicrobial resistance. Lancet Infect. Dis. 23:e281. doi: 10.1016/S1473-3099(23)00446-2, PMID: 37516131

[ref48] Johnsen P. J. Townsend J. P. Bøhn T. Simonsen G. S. Sundsfjord A. Nielsen K. M. (2009). Factors affecting the reversal of antimicrobial-drug resistance. Lancet Infect. Dis. 9, 357–364. doi: 10.1016/S1473-3099(09)70105-7, PMID: 19467475

[ref49] Kaper J. B. Nataro J. P. Mobley H. L. (2004). Pathogenic *Escherichia coli*. Nat. Rev. Microbiol. 2, 123–140. doi: 10.1038/nrmicro818, PMID: 15040260

[ref50] Kawamura K. Goto K. Nakane K. Arakawa Y. (2014). Molecular epidemiology of extended-spectrum β-lactamases and *Escherichia coli* isolated from retail foods including chicken meat in Japan. Foodborne Pathog. Dis. 11, 104–110. doi: 10.1089/fpd.2013.1608, PMID: 24093132

[ref51] Keck N. Treilles M. Gordoncillo M. Ivette O. L. I. Dauphin G. Dorado-Garcia A.. (2023). A systematic approach toward progressive improvement of national antimicrobial resistance surveillance systems in food and agriculture sectors. Front. Vet. Sci. 9:1057040. doi: 10.3389/fvets.2022.1057040, PMID: 36825205 PMC9941986

[ref52] Kelbert L. Barmettler K. Biggel M. Stephan R. Nüesch-Inderbinen M. (2025). Occurrence and characteristics of extended-spectrum ß-lactamase-producing *Escherichia coli* in Swiss and imported retail chicken meat. J. Glob. Antimicrob. Resist. 43, 285–292. doi: 10.1016/j.jgar.2025.05.013, PMID: 40381802

[ref53] Kim Y. J. Moon J. S. Oh D. H. Chon J. W. Song B. R. Lim J. S.. (2018). Genotypic characterization of ESBL-producing *E. coli* from imported meat in South Korea. Food Res. Int. 107, 158–164. doi: 10.1016/j.foodres.2017.12.023, PMID: 29580473

[ref54] Klein H. Vidal F. (2022). The emergence of Brazil as the leading world exporter of chicken meat. Hist. Agrar. Am. Lat. 3, 75–99. doi: 10.53077/haal.v3i02.127

[ref55] Leigue L. Warth J. F. Melo L. C. Silva K. C. Moura R. A. Barbato L.. (2015). MDR ST2179-CTX-M-15 *Escherichia coli* co-producing RmtD and AAC(6′)-Ib-cr in a horse with extraintestinal infection, Brazil. J. Antimicrob. Chemother. 70, 1263–1265. doi: 10.1093/jac/dku520, PMID: 25538170

[ref56] Li Q. Li Z. Wang Y. Chen Y. Sun J. Yang Y.. (2022). Antimicrobial resistance and transconjugants characteristics of *sul3* positive *Escherichia coli* isolated from animals in Nanning, Guangxi Province. Animals 12:976. doi: 10.3390/ani12080976, PMID: 35454223 PMC9025041

[ref57] Li Q. Zou H. Wang D. Zhao L. Meng M. Wang Z.. (2023). Tracking spatio-temporal distribution and transmission of antibiotic resistance in aquatic environments by using ESBL-producing *Escherichia coli* as an indicator. J. Environ. Manag. 344:118534. doi: 10.1016/j.jenvman.2023.118534, PMID: 37393874

[ref58] Liu H. Fan S. Zhang X. Yuan Y. Zhong W. Wang L.. (2024). Antibiotic-resistant characteristics and horizontal gene transfer ability analysis of extended-spectrum β-lactamase-producing *Escherichia coli* isolated from giant pandas. Front. Vet. Sci. 11:1394814. doi: 10.3389/fvets.2024.1394814, PMID: 39132438 PMC11310934

[ref59] Magiorakos A. P. Srinivasan A. Carey R. B. Carmeli Y. Falagas M. E. Giske C. G.. (2012). Multidrug-resistant, extensively drug-resistant and pandrug-resistant bacteria: an international expert proposal for interim standard definitions for acquired resistance. Clin. Microbiol. Infect. 18, 268–281. doi: 10.1111/j.1469-0691.2011.03570.x, PMID: 21793988

[ref60] Manges A. R. Smith S. P. Lau B. J. Nuval C. J. Eisenberg J. N. Dietrich P. S.. (2007). Retail meat consumption and the acquisition of antimicrobial resistant *Escherichia coli* causing urinary tract infections: a case-control study. Foodborne Pathog. Dis. 4, 419–431. doi: 10.1089/fpd.2007.0026, PMID: 18041952

[ref61] Martínez-Álvarez S. Châtre P. François P. Zarazaga M. Madec J. Y. Haenni M.. (2025). Comparative phylogenomics of extended-spectrum beta-lactamase-producing *Escherichia coli* revealed a wide diversity of clones and plasmids in Spanish chicken meat. Int. J. Food Microbiol. 426:110900. doi: 10.1016/j.ijfoodmicro.2024.110900, PMID: 39305653

[ref62] McEwen S. A. Collignon P. J. (2018). Antimicrobial resistance: a one health perspective. Microbiol. Spectr. 6. doi: 10.1128/microbiolspec.arba-0009-2017 PMC11633550 29600770

[ref63] Millman J. M. Waits K. Grande H. Marks A. R. Marks J. C. Price L. B.. (2013). Prevalence of antibiotic-resistant *E. coli* in retail chicken: comparing conventional, organic, kosher, and raised without antibiotics. F1000Res 2:155. doi: 10.12688/f1000research.2-155.v2, PMID: 24555073 PMC3901448

[ref64] Mo S. S. Fiskebeck E. Z. Slettemeås J. S. Lagesen K. Nilsson O. Naseer U.. (2023). *Escherichia coli* multilocus sequence type 38 from humans and broiler production represent distinct monophyletic groups. Front. Microbiol. 14:1173287. doi: 10.3389/fmicb.2023.1173287, PMID: 37266008 PMC10231635

[ref65] Mohammadi H. Saghaian S. Boccia F. (2023). Antibiotic-free poultry meat consumption and its determinants. Food Secur. 12:1776. doi: 10.3390/foods12091776, PMID: 37174314 PMC10177776

[ref66] Mosquito S. Pons M. J. Riveros M. Ruiz J. Ochoa T. J. (2015). Diarrheagenic *Escherichia coli* phylogroups are associated with antibiotic resistance and duration of diarrheal episode. Sci. World J. 2015. doi: 10.1155/2015/610403, PMID: 25811044 PMC4355820

[ref67] Mughini-Gras L. Dorado-García A. van Duijkeren E. van den Bunt G. Dierikx C. M. Bonten M. J. M.. (2019). Attributable sources of community-acquired carriage of *Escherichia coli* containing β-lactam antibiotic resistance genes: a population-based modelling study. Lancet Planet Health 3, e357–e369. doi: 10.1016/S2542-5196(19)30130-5, PMID: 31439317

[ref68] Müller A. Stephan R. Nüesch-Inderbinen M. (2016). Distribution of virulence factors in ESBL-producing *Escherichia coli* isolated from the environment, livestock, food and humans. Sci. Total Environ. 541, 667–672. doi: 10.1016/j.scitotenv.2015.09.135, PMID: 26437344

[ref69] Murray M. Salvatierra G. Dávila-Barclay A. Ayzanoa B. Castillo-Vilcahuaman C. Huang M.. (2021). Market chickens as a source of antibiotic-resistant *Escherichia coli* in a Peri-Urban Community in Lima, Peru. Front. Microbiol. 12:635871. doi: 10.3389/fmicb.2021.635871, PMID: 33737922 PMC7961087

[ref70] Nahar A. Awasthi S. P. Hatanaka N. Okuno K. Hoang P. H. Hassan J.. (2018). Prevalence and characteristics of extended-spectrum β-lactamase-producing *Escherichia coli* in domestic and imported chicken meats in Japan. J. Vet. Med. Sci. 80, 510–517. doi: 10.1292/jvms.17-0708, PMID: 29434117 PMC5880835

[ref71] Oikarainen P. E. Pohjola L. K. Pietola E. S. Heikinheimo A. (2019). Direct vertical transmission of ESBL/pAmpC-producing *Escherichia coli* limited in poultry production pyramid. Vet. Microbiol. 231, 100–106. doi: 10.1016/j.vetmic.2019.03.001, PMID: 30955795

[ref72] Palmeira J. D. Ferreira H. Madec J. Y. Haenni M. (2018). Draft genome of a ST443 *mcr-1*- and *bla*_CTX-M-2_-carrying *Escherichia coli* from cattle in Brazil. J. Glob. Antimicrob. Resist. 13, 269–270. doi: 10.1016/j.jgar.2018.05.010, PMID: 29800745

[ref73] Park J. H. Kim H. S. Yim J. H. Kim Y. J. Kim D. H. Chon J. W.. (2017). Comparison of the isolation rates and characteristics of *Salmonella* isolated from antibiotic-free and conventional chicken meat samples. Poult. Sci. 96, 2831–2838. doi: 10.3382/ps/pex055, PMID: 28482031

[ref74] Parvin M. S. Talukder S. Ali M. Y. Chowdhury E. H. Rahman M. T. Islam M. T. (2020). Antimicrobial resistance pattern of *Escherichia coli* isolated from frozen chicken meat in Bangladesh. Pathogens 9:420. doi: 10.3390/pathogens9060420, PMID: 32481680 PMC7350304

[ref75] Plaza-Rodríguez C. Mesa-Varona O. Alt K. Grobbel M. Tenhagen B. A. Kaesbohrer A. (2021). Comparative analysis of consumer exposure to resistant Bacteria through chicken meat consumption in Germany. Microorganisms 9:1045. doi: 10.3390/microorganisms9051045, PMID: 34066213 PMC8151568

[ref76] Puljko A. Babić I. Rozman S. D. Barišić I. Jelić M. Maravić A.. (2024). Treated municipal wastewater as a source of high-risk and emerging multidrug-resistant clones of *E. Coli* and other Enterobacterales producing extended-spectrum β-lactamases. Environ. Res. 243:117792. doi: 10.1016/j.envres.2023.117792, PMID: 38048868

[ref77] Ramos S. Silva V. Dapkevicius M. L. E. Caniça M. Tejedor-Junco M. T. Igrejas G.. (2020). *Escherichia coli* as commensal and pathogenic bacteria among food-producing animals: health implications of extended spectrum β-lactamase (ESΒL) production. Animals 10:2239. doi: 10.3390/ani10122239 33260303 PMC7761174

[ref78] Rawat N. Anjali S. Sabu B. Bandyopadhyay A. Rajagopal R. (2024). Assessment of antibiotic resistance in chicken meat labelled as antibiotic-free: a focus on *Escherichia coli* and horizontally transmissible antibiotic resistance genes. LWT 194:115751. doi: 10.1016/j.lwt.2024.115751

[ref79] Saidenberg A. B. S. Edslev S. M. Hallstrøm S. Rasmussen A. Park D. E. Aziz M.. (2024). *Escherichia coli* ST117: exploring the zoonotic hypothesis. Microbiol. Spectr. 3:e0046624. doi: 10.1128/spectrum.00466-24 PMC11448156 39235965

[ref9001] Sanchez H. M. Whitener V. A. Thulsiraj V. Amundson A. Collins C. Duran-Gonzalez M.. (2020). Antibiotic Resistance of Escherichia coli Isolated from Conventional, No Antibiotics, and Humane Family Owned Retail Broiler Chicken Meat. Animals (Basel). 26:2217. doi: 10.3390/ani10122217 PMC7760345 33256102

[ref80] Sarkar S. Souza M. J. Martin-Jimenez T. Abouelkhair M. A. Kania S. A. Okafor C. C. (2023). Tetracycline, sulfonamide, and erythromycin residues in beef, eggs, and honey sold as "antibiotic-free" products in East Tennessee (USA) Farmers' Markets. Vet. Sci. 10:243. doi: 10.3390/vetsci10040243, PMID: 37104399 PMC10143955

[ref81] Seni J. Moremi N. Matee M. van der Meer F. DeVinney R. Mshana S. E.. (2018). Preliminary insights into the occurrence of similar clones of extended-spectrum beta-lactamase-producing bacteria in humans, animals and the environment in Tanzania: a systematic review and meta-analysis between 2005 and 2016. Zoonoses Public Health 65, 1–10. doi: 10.1111/zph.12387, PMID: 28834351

[ref9002] Singer R. S. Porter L. J. Thomson D. U. Gage M. Beaudoin A. Wishnie J. K. (2019). Raising Animals Without Antibiotics: U.S. Producer and Veterinarian Experiences and Opinions. Front Vet Sci. 6:452. doi: 10.3389/fvets.2019.00452 31867349 PMC6910073

[ref82] Singhal N. Kumar M. Kanaujia P. K. Virdi J. S. (2015). MALDI-TOF mass spectrometry: an emerging technology for microbial identification and diagnosis. Front. Microbiol. 6:791. doi: 10.3389/fmicb.2015.00791, PMID: 26300860 PMC4525378

[ref83] Soncini J. G. M. Cerdeira L. Sano E. Koga V. L. Tizura A. T. Tano Z. N.. (2022). Genomic insights of high-risk clones of ESΒL-producing *Escherichia coli* isolated from community infections and commercial meat in southern Brazil. Sci. Rep. 12:9354. doi: 10.1038/s41598-022-13197-y, PMID: 35672430 PMC9174282

[ref84] Sun Z. Hong W. Xue C. Dong N. (2024). A comprehensive review of antibiotic resistance gene contamination in agriculture: challenges and AI-driven solutions. Sci. Total Environ. 953:175971. doi: 10.1016/j.scitotenv.2024.175971, PMID: 39236811

[ref85] Tafoukt R. Touati A. Leangapichart T. Bakour S. Rolain J. M. (2017). Characterization of OXA-48-like-producing Enterobacteriaceae isolated from river water in Algeria. Water Res. 120, 185–189. doi: 10.1016/j.watres.2017.04.073, PMID: 28486169

[ref86] Tang K. L. Caffrey N. P. Nóbrega D. B. Cork S. C. Ronksley P. E. Barkema H. W.. (2019). Comparison of different approaches to antibiotic restriction in food-producing animals: stratified results from a systematic review and meta-analysis. BMJ Glob. Health 4:e001710. doi: 10.1136/bmjgh-2019-001710, PMID: 31543995 PMC6730577

[ref87] Thanner S. Drissner D. Walsh F. (2016). Antimicrobial resistance in agriculture. MBio 7:e02227. doi: 10.1128/mBio.02227-15 27094336 PMC4850276

[ref88] Tian M. He X. Feng Y. Wang W. Chen H. Gong M.. (2021). Pollution by antibiotics and antimicrobial resistance in LiveStock and poultry manure in China, and countermeasures. Antibiotics 10:539. doi: 10.3390/antibiotics10050539, PMID: 34066587 PMC8148549

[ref89] U.S. Food and Drug Administration (2021). National antimicrobial resistance monitoring system (NARMS): retail meat surveillance laboratory protocol. Maryland: FDA.

[ref90] van Hoek A. H. A. M. Veenman C. Florijn A. Huijbers P. M. C. Graat E. A. M. de Greeff S.. (2018). Longitudinal study of ESΒL *Escherichia coli* carriage on an organic broiler farm. J. Antimicrob. Chemother. 73, 3298–3304. doi: 10.1093/jac/dky362 30219829

[ref91] Warriner K. Aldsworth T. G. Kaur S. Dodd C. E. (2002). Cross-contamination of carcasses and equipment during pork processing. J. Appl. Microbiol. 93, 169–177. doi: 10.1046/j.1365-2672.2002.01678.x, PMID: 12067387

[ref92] World Health Organization (2017). Stop using antibiotics in healthy animals to prevent the spread of antibiotic resistance. Geneva: World Health Organization.

[ref93] World Health Organization (2024). WHO bacterial priority pathogens list, 2024: bacterial pathogens of public health importance to guide research, development and strategies to prevent and control antimicrobial resistance. Geneva: World Health Organization.

[ref9003] Yu Z. Wang Q. Pinilla-Redondo R. Stenløkke Madsen J. Anna Dam Clasen K. Ananbeh H.. (2024). Horizontal transmission of a multidrug-resistant IncN plasmid isolated from urban wastewater. Ecotoxicol Environ Saf. 271:115971. doi: 10.1016/j.ecoenv.2024.115971 38237397

