# Supplementary figures and images for: Extended-spectrum β-lactamase (ESβL)-producing Escherichia coli in antibiotic-free and conventional chicken meat, Brazil
Source: Front Microbiol. 2025 Jul 8;16:1593887. doi: 10.3389/fmicb.2025.1593887 (PMC12279825; doi:10.3389/fmicb.2025.1593887)

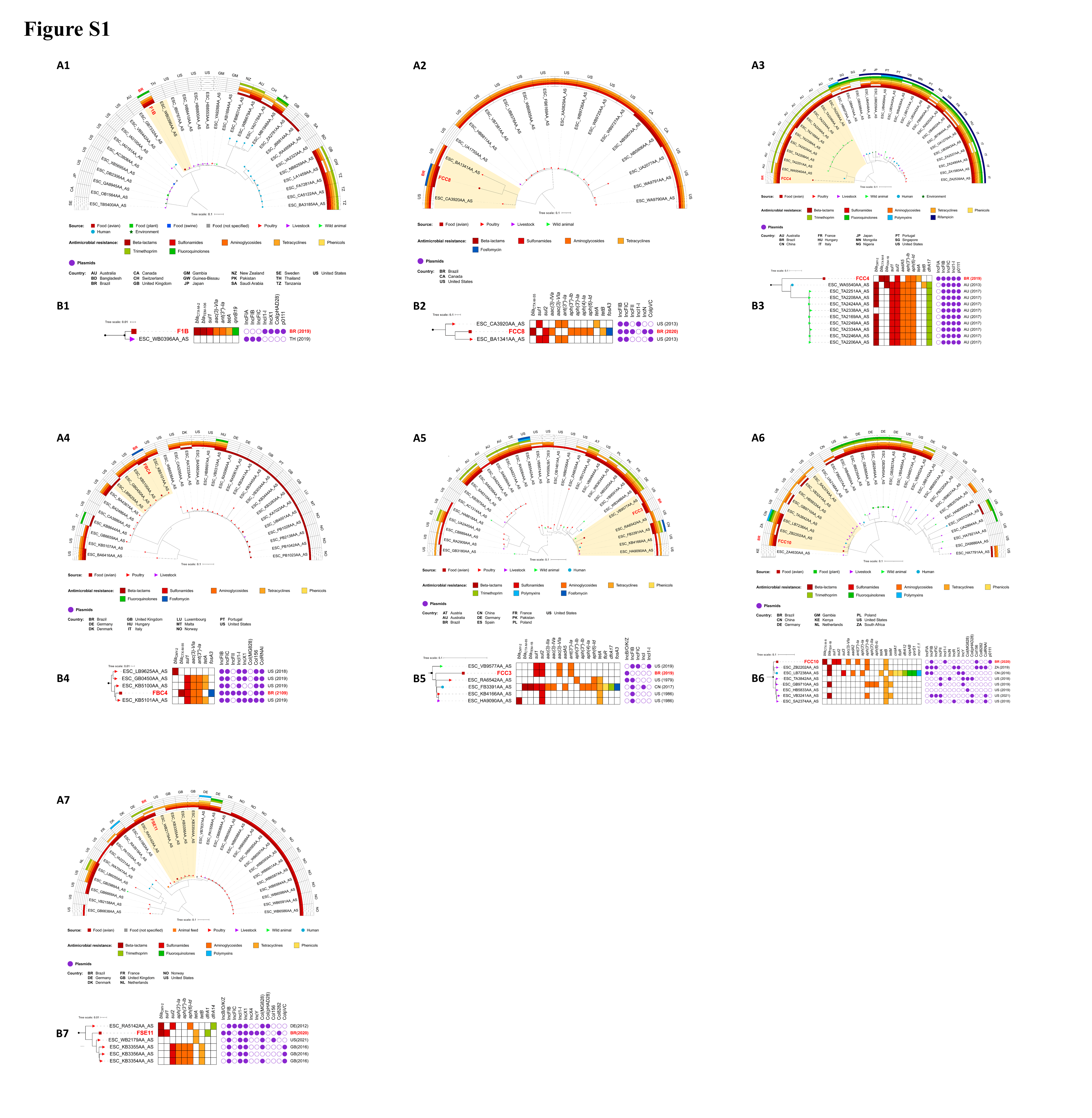

Supplement: SUPPLEMENTARY FIGURE S1 — Phylogenetic analysis. In A1–A7, maximum-likelihood phylogenetic tree of F1B (ST443), FCC8 (ST3258), FCC4 (ST2179), FBC4 (ST2040), FCC3 (ST350), FCC10 (ST15579), and FSE11 (ST57) Escherichia coli strains. Comparison of resistomes, isolation sources, and countries of origin of the strains. In B1–B7, zooming into the subtree comprising sequenced strains of Escherichia coli in this study. The figure was generated with iTOL version 5.6.1 (https://itol.embl.de). [file Image_1.TIF]
